# Polymer-Tethered Quenched Fluorescent Probes for Enhanced Imaging of Tumor-Associated Proteases

**DOI:** 10.1021/acssensors.4c00912

**Published:** 2024-06-28

**Authors:** Martin Hadzima, Franco F. Faucher, Kristýna Blažková, Joshua J. Yim, Matteo Guerra, Shiyu Chen, Emily C. Woods, Ki Wan Park, Pavel Šácha, Vladimír Šubr, Libor Kostka, Tomáš Etrych, Pavel Majer, Jan Konvalinka, Matthew Bogyo

**Affiliations:** † Institute of Organic Chemistry and Biochemistry, Czech Academy of Sciences, Flemingovo n. 2, Praha 6 16610, Czech Republic; ‡ Department of Organic Chemistry, Faculty of Science, Charles University, Albertov 6, Praha 2 12800, Czech Republic; § Department of Chemistry, Stanford University, Stanford, California 94305, United States; ∥ Department of Pathology, School of Medicine, Stanford University, Stanford, California 94305, United States; ⊥ Institute of Macromolecular Chemistry, Czech Academy of Sciences, Heyrovského n. 2, Praha 6 16206, Czech Republic

**Keywords:** fluorescence, imaging, protease, cancer, HPMA copolymer, iBody

## Abstract

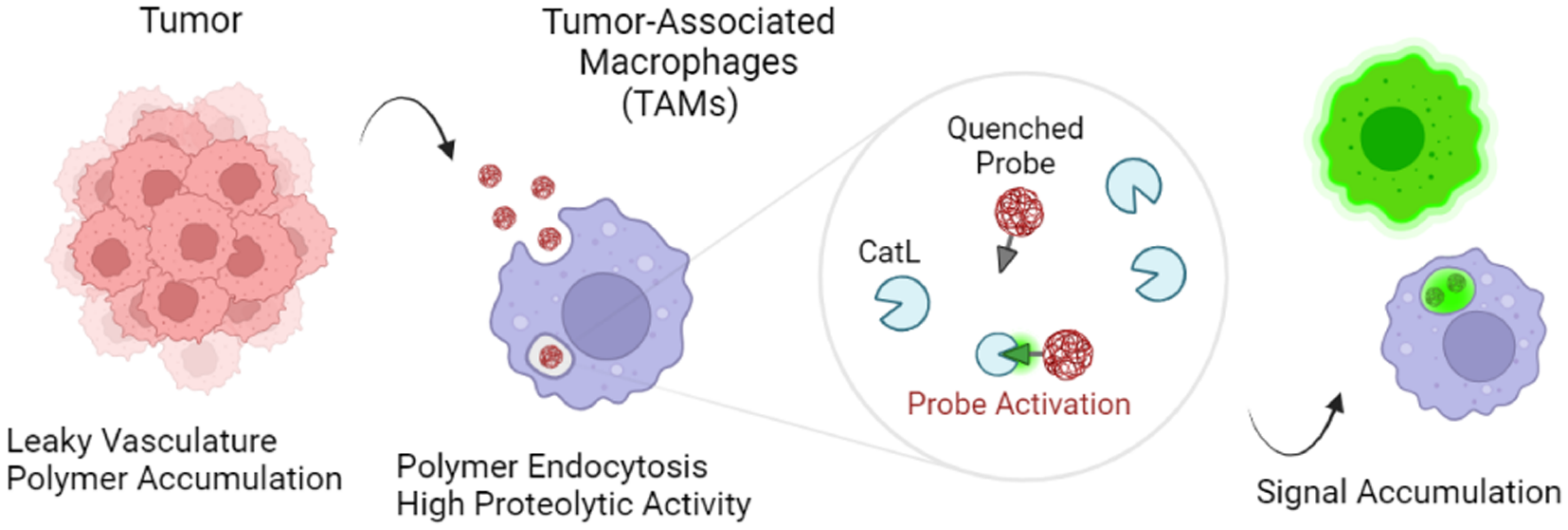

Fluorescence-based contrast agents enable real-time detection of solid tumors and their neovasculature, making them ideal for use in image-guided surgery. Several agents have entered late-stage clinical trials or secured FDA approval, suggesting they are likely to become the standard of care in cancer surgeries. One of the key parameters to optimize in contrast agents is molecular size, which dictates much of the pharmacokinetic and pharmacodynamic properties of the agent. Here, we describe the development of a class of protease-activated quenched fluorescent probes in which a *N*-(2-hydroxypropyl)methacrylamide copolymer is used as the primary scaffold. This copolymer core provides a high degree of probe modularity to generate structures that cannot be achieved with small molecules and peptide probes. We used a previously validated cathepsin substrate and evaluated the effects of length and type of linker, as well as the positioning of the fluorophore/quencher pair on the polymer core. We found that the polymeric probes could be optimized to achieve increased overall signal and tumor-to-background ratios compared to the reference small molecule probe. Our results also revealed multiple structure–activity relationship trends that can be used to design and optimize future optical imaging probes. Furthermore, they confirm that a hydrophilic polymer is an ideal scaffold for use in optical imaging contrast probes, allowing a highly modular design that enables efficient optimization to maximize probe accumulation and overall biodistribution properties.

Precise tumor resection during surgical procedures remains a challenge due to difficulties with the detection of tumor margins in real-time with high specificity and accuracy. Fluorescent probes have emerged as powerful tools with the potential to revolutionize cancer treatment as they provide contrast without the need for exposure to radiation.^1−5^ Furthermore, the U.S. Food and Drug Administration (FDA) approval of multiple fluorescence imaging systems for use during surgery has created an opportunity to rapidly implement new optical contrast agents into existing surgical workflows. While FDA-approved fluorescent agents such as indocyanine green^5,6^ and methylene blue^5,7^ can be used in high doses to visualize some types of solid tumors through passive uptake or exclusion of the free dyes, their imaging performance is limited by the absence of a specific targeting mechanism. Recently, the FDA approved the first affinity-based fluorescent probe for cancer imaging, the OTL38 (CYTALUX).^8,9^ OTL38 is a folic acid derivative bearing a near-infrared (NIR) fluorophore that exhibits high affinity for folate receptor α (FRα), which is often overexpressed in multiple types of solid tumors. In addition, activity-based probes, relying on specific enzymatic activation within the target tissue, are now reaching late-stage clinical trials.^10,11^ Examples include the PEGylated peptidic substrate LUM015^10,12^ and the irreversible covalent probe VGT-309,^11,13^ both targeting cathepsins. While fluorescent probes have already begun to demonstrate significant value for surgical guidance, there remains a need for further strategies to improve key parameters such as tumor-to-background signal ratio (TBR), tissue selectivity, and overall signal half-life.

One strategy to increase circulation time and tumor retention, resulting in enhanced TBR, involves increasing molecular weight and hydrophilicity. The use of PEGylation to increase probe size is a common design principle that leverages the enhanced permeability and retention (EPR) effect commonly observed in malignant tissues.^14,15^ One of the first enzyme-activated optical probes, introduced by Weissleder et al.,^16^ was a self-quenched PEGylated poly lysine copolymer with a molecular weight close to 500 kDa. The cathepsin-activated probe LUM015, currently under clinical investigation, also exploits a large PEG-based scaffold. We hypothesized that a hydrophilic, inert, and biocompatible macromolecular backbone, combined with an activity-based fluorescent substrate, could yield a probe with enhanced performance compared to its small-molecular counterparts. Furthermore, the macromolecular scaffold can be used to attach additional affinity-based targeting elements and control the overall location and stoichiometry of quencher-fluorophore pairs. A recently developed class of macromolecular antibody mimetics, called iBodies, employs a platform based on *N*-(2-hydroxypropyl)methacrylamide copolymer (pHPMA).^17^ The concept of iBodies is based on facile derivatization in the polymer side chains with high-affinity ligands or inhibitors, fluorescent dyes, and/or appropriate affinity tags, such as biotin, all on the same pHPMA carrier. Modular iBodies are similar to antibodies with high affinity but with increased overall functionality and design flexibility, similar to synthetic small molecules. iBody conjugates can be used for both imaging and inhibition of enzymes, and the pHPMA carrier enables control of the density of displayed ligands to increase binding affinity and inhibitory potency.^18−22^ This platform has also been applied for the specific binding of His-tagged proteins.^23^ Based on the success of iBodies, we hypothesized that pHPMA could be a promising template for improving the biodistribution and contrast of low-molecular weight protease-activated contrast agents. In this study, we describe macromolecular quenched fluorescent probes based on pHPMA. We used this carrier to investigate the effects of quencher-fluorophore positioning and stoichiometry, as well as linker choice, on overall probe performance in a mouse model of breast cancer. The pHPMA probes showed superior performance and biodistribution compared to the reference small molecular contrast agent. Furthermore, we found that their performance was dependent on the choice of linker and the structure of individual probes, providing a basis for the design and optimization of polymer-based quenched probes for targeting cancer and other diseases.

## Results

### Design of the Macromolecular Probes and Their Precursors

**Figure 1:**
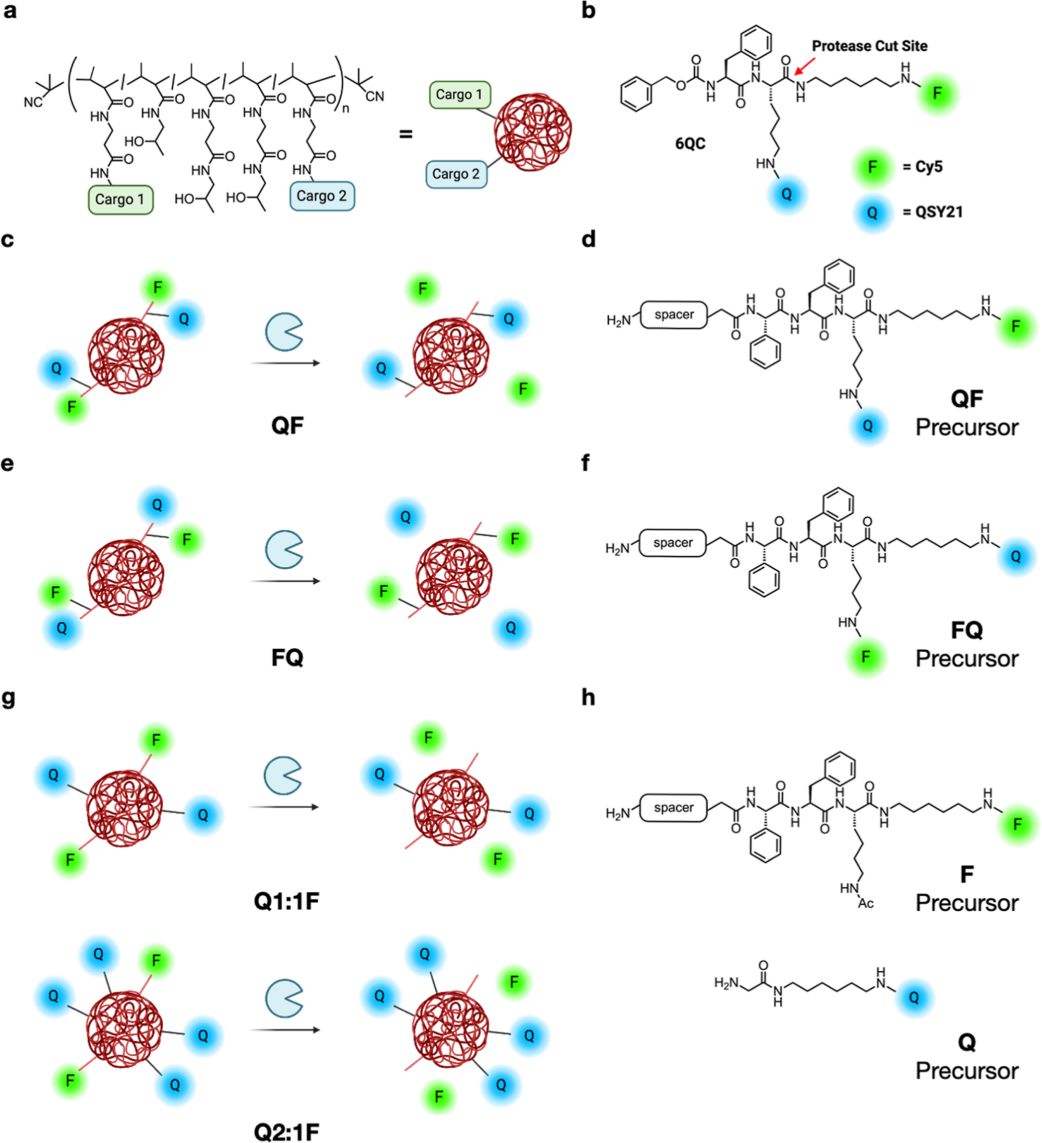
Chemical structure of employed *N*-(2-hydroxypropyl)methacrylamide copolymer (pHPMA) and probe arrangements. (a) Schematic representation of iBodies. Copolymer side chains are modified with various functional moieties via amide coupling (Cargo 1 and Cargo 2). Abundance and diversity of individual moieties can be controlled during pHPMA precursor modification. (b) Structure of the parental small molecule probe **6QC** with protease cut site highlighted by red arrow. In this study, Q (blue sphere) represents sulfo-QSY21, while F (green sphere) represents sulfo-Cy5. (c–g) Proposed arrangements of probe on the polymer or “architectures” (c) QF architecture where cleavage releases the fluorophore from the polymer, (d) structure of the ligand used in the QF architecture, (e) FQ architecture where cleavage releases the quencher from the polymer, (f) structure of the FQ ligand, (g) Q1:1F and Q2:1F structures where quencher and fluorophore are attached separately at controlled ratios and cleavage releases the fluorophore from the polymer, and (h) structure of Q and F ligands for the Q*x*:1F architecture. Created with BioRender.com.

We proposed that macromolecular probes derived from “iBodies” (Figure 1a) would offer distinct advantages over their small-molecule counterparts by improving the overall biodistribution while leveraging the synthetic flexibility of a biocompatible hydrophilic backbone. The pHPMA precursors P1–P3 (Table S1) with controlled molecular weight and low dispersity were prepared by the reversible addition–fragmentation chain transfer (RAFT) copolymerization of HPMA and 3-(3-methacrylamidopropanoyl)thiazolidine-2-thione (Ma-β-Ala-TT). The thiazolidine-2-thione (TT) reactive groups along the pHPMA chain enable the attachment of amine-terminated low-molecular weight contrast agents. Because multiple different components can be built into the pHPMA at controlled ratios, it is possible to regulate the abundancy of each moiety on the carrier, allowing fine-tuning to optimize overall signal intensity. As a starting point for this study, we chose a previously published quenched pan-cathepsin substrate **6QC**, containing sulfo-Cy5 as the fluorophore and sulfo-QSY21 as the quencher (Figure 1b).^24^ Cathepsin-targeted probes have proven to be valuable tools for tumor imaging^12,13,25−27^ and **6QC**, in particular, has been optimized for this application.^24,28,29^ We designed analogues of **6QC** that introduce a spacer and a terminal amino group that can be used for incorporation onto the pHPMA precursor (Figure 1d,f,h). We have previously shown that the N-terminal benzyloxycarbonyl (Cbz) group plays an important role in the substrate recognition of **6QC**;^27^ therefore, we substituted the Cbz group with phenylglycine to preserve this motif in the spacer-modified analogues, which enabled the N-terminus to serve as a handle for spacer attachment. We designed several “architectures” available on the macromolecular carrier. In the primary probe design, we preserved the quencher-fluorophore arrangement of the original **6QC** fragment, where the two are positioned on the substrate peptide backbone (Figure 1d). In the case of the QF probe, protease cleavage releases the fluorophore from the pHPMA carrier as a free amine, which can be protonated and retained in the acidic lysosomes of macrophages (Figure 1c). We then inverted the positions of the fluorophore and the quencher to generate the FQ probe (Figure 1e,f). This configuration results in the release of the quencher by the protease, leaving the fluorophore tethered to the pHPMA carrier. In addition, the tunable iBodies-based platform enables separation of the quencher and the fluorophore by tethering the two components to the pHPMA carrier separately (Figure 1g). In this case, we attached the quencher to the pHPMA via a short non-cleavable linker, while the fluorophore was attached using the cathepsin-cleavable substrate fragment (Figure 1h). The iBody platform also enables precise control over the quencher-fluorophore ratio during synthesis. In this way, we can investigate how a change in the ratio of quenchers to fluorophores can affect the imaging properties of the probes.

For our initial studies, we generated pHPMA probes with one-to-one (Q1:1F), two-to-one (Q2:1F), and three-to-one (Q3:1F) quencher-fluorophore ratios. Building upon this design, we synthesized a set of pHPMA probes to investigate the impact of the spacer and the architecture on the probe performance.

### *In Vitro* Evaluation of Probe Cleavage by Recombinant Cathepsin L and in RAW 264.7 Mouse Macrophages

**Figure 2:**
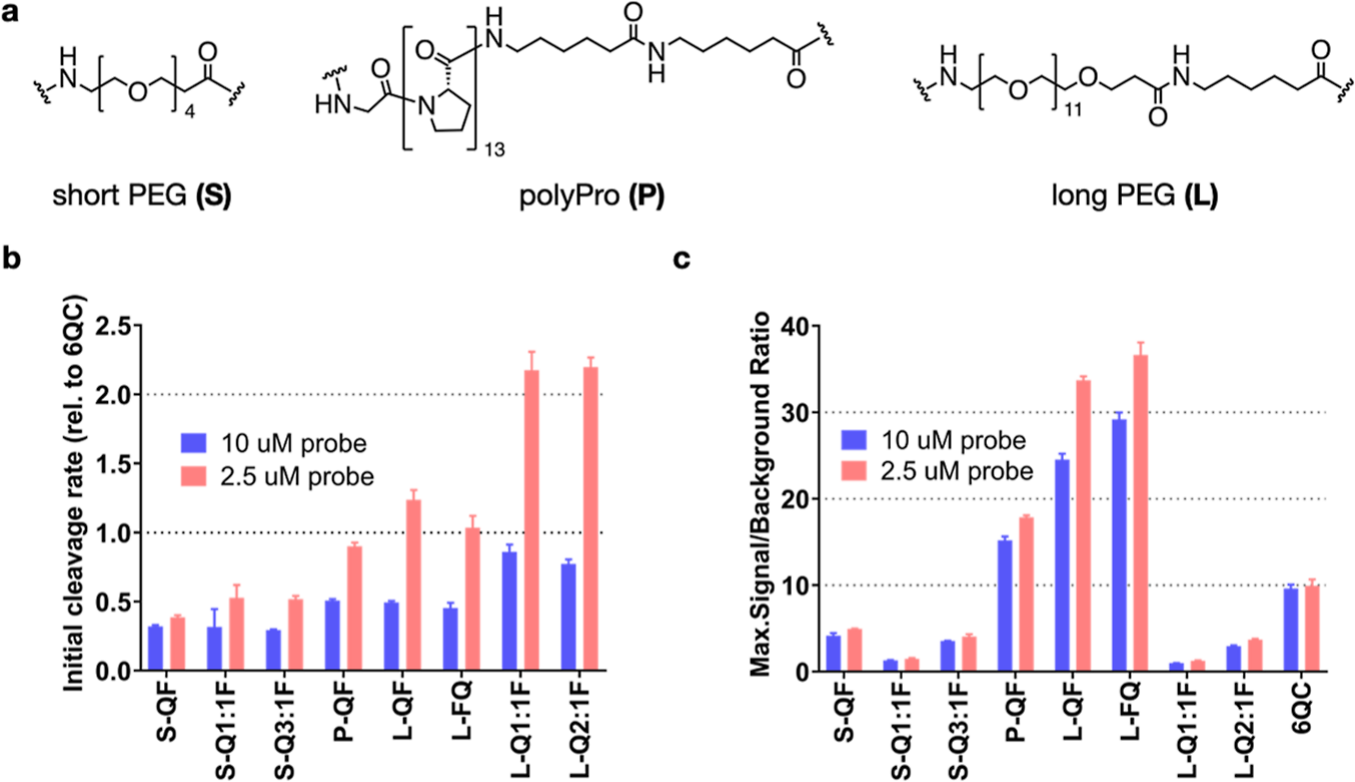
Effect of linker and architecture on *in vitro* probe activation by cathepsin L (CatL) (a) Structures of employed linkers. Polymer compositions and characteristics can be found in Table S2. (b) Bar graph showing initial cleavage rate of selected probes by purified CatL. Rates were measured at 2 probe concentrations [10, 2.5 μM] and normalized to **6QC** as a standard. (c) Bar graph showing maximal signal-to-background ratio (SBR) calculated from the cleavage assay as a ratio between end point signal (90 min) in positive (CatL) and negative (no CatL) samples. SBR was measured at 2 concentrations [10, 2.5 μM]. All experiments were performed in 96-well plates in triplicates. Data were collected over 90 min using a Biotek Cytation 3 plate reader. Error bars represent standard deviation. All values can be found in Table S3.

We employed three different linkers: a short and flexible polyethylene glycol linker (PEG-4, referred to as short, S), a more rigid polyproline linker (13 prolines, polyPro, P), and a longer version of the PEG linker (PEG-12, referred to as long, L) (Figure 2a). The detailed compositions and characteristics of the pHPMA probes can be found in Table S2. We assessed the efficiency of purified cathepsin L (CatL) to cleave our macromolecular probes at two different probe concentrations (10, 2.5 μM; to reduce the influence of concentration-related effects such as aggregation of the macromolecular conjugates; Table S3). These results confirmed that the cleavage rate was highly dependent on linker length and was only marginally influenced by probe architecture (Figure 2b). Probes **S-QF**, **S-Q1:1F**, and **S-Q3:1F** were cleaved at a slower rate than **6QC** at both 10 and 2.5 μM. The polyPro probe **P-QF** was cleaved more efficiently than the **S-QF** probe with the initial cleavage rate at 2.5 μM almost reaching that of **6QC**. Probes **L-QF**, **L-FQ**, **L-Q1:1F**, and **L-Q2:1F** had the highest cleavage rates, exceeding that of **6QC** at 2.5 μM, and reaching a relative rate of 2.20 ± 0.07 for **L-Q2:1F** (Table S3), suggesting that the rigidity of the polyPro linker did not yield a significant benefit compared to the flexible PEG-12. All pHPMA probes were cleaved at a higher relative rate at the lower probe concentration of 2.5 μM. We also analyzed the maximal signal-to-background ratio (SBR) of individual probes in the assay (Figure 2c). This value is dependent on both the cleavage efficiency as well as the quenching efficiency, which is influenced by the quencher-fluorophore distance and ratio on the probe. For all of the polymeric probes, we used fluorophore loadings of approximately two units per polymer chain (Table S2). The short PEG series probe **S-QF** had a lower SBR than **P-QF** or **L-QF** due to less efficient cleavage. However, we also observed a further drop in SBR values for **S-Q1:1F** and **S-Q3:1F** probes that bear the quencher on the backbone (all values can be found in Table S3). This is likely due to the increased quencher-fluorophore distance when the quencher is positioned on the pHPMA backbone. Swapping the positions of sulfo-Cy5 and sulfo-QSY21 in the **L-FQ** probe did not influence the cleavage rate or the quenching efficiency significantly. Analogous to the short linker probes, the long linker probes **L-Q1:1F** and **L-Q2:1F** exhibited lower SBR than **L-QF**. Of all the probes tested, **L-QF** and **L-FQ** reached the highest SBR values, with an over 3-fold increase compared to **6QC**, likely due to efficient cleavage, quenching, and the presence of multiple fluorophores on the pHPMA carrier. The Q1:1F architecture showed the lowest SBR in the individual series, while the addition of quencher moieties to the polymer led to an increase in SBR but never reached the values observed with QF/FQ architecture.

**Figure 3:**
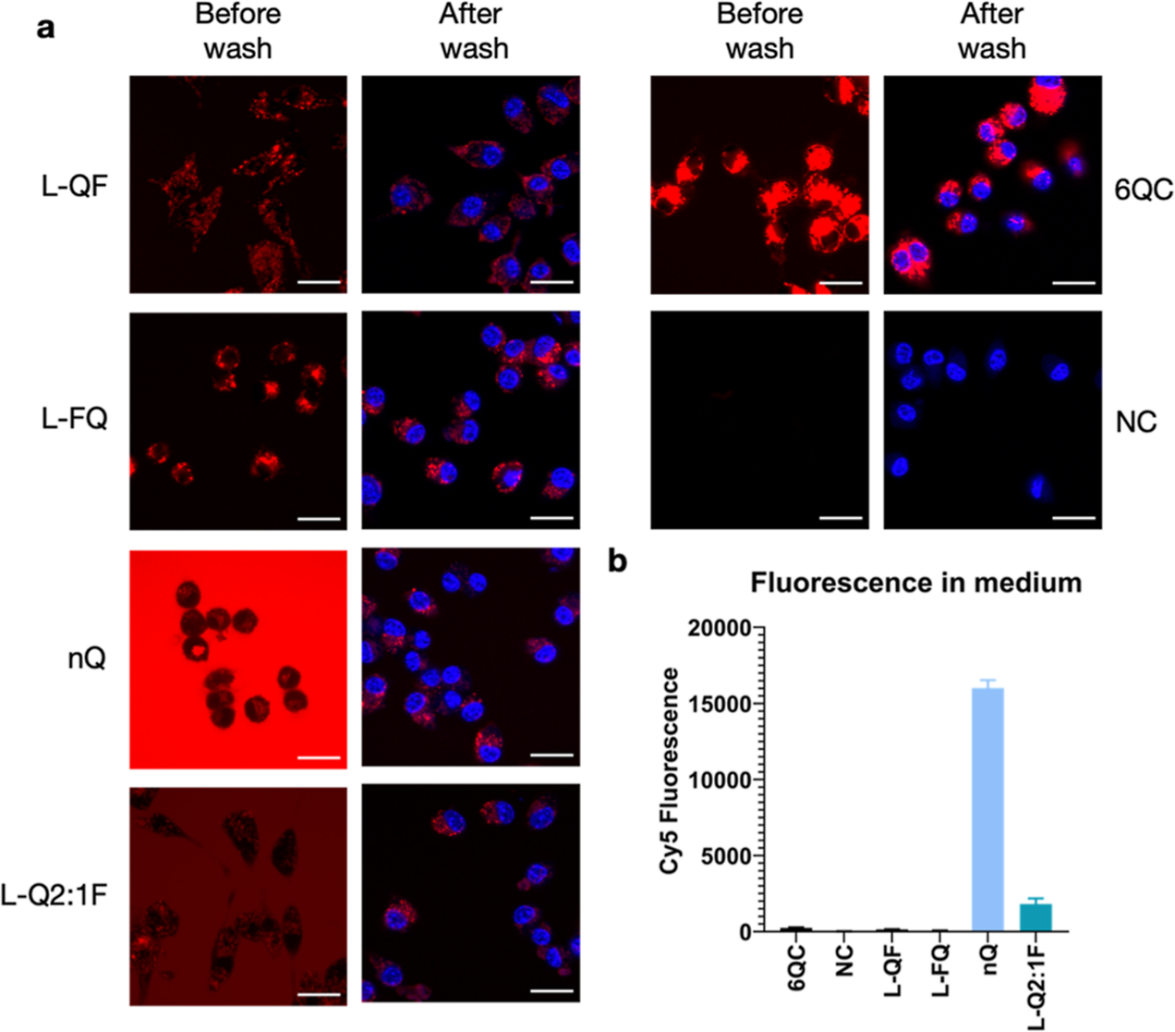
Internalization and cleavage of probes in RAW 264.7 mouse macrophages. (a) Confocal microscopy images of RAW 264.7 cells incubated with the indicated probes for 2 h at 37 °C. Cells were imaged directly after 2 h (first column) or washed and stained with Hoechst 33342 (second column). Imaging was performed with excitation at 639 nm and emission at 669 nm for Cy5 (colored red) and excitation at 405 nm and emission at 435 nm for Hoechst (colored blue). Scale bar = 20 μm. (b) Bar graph showing average Cy5 fluorescence intensity in conditioned medium after 2 h of incubation. Data is present for four experiments. Error bars represent standard deviation.

To monitor the internalization and cleavage of selected pHPMA probes in comparison with the reference **6QC** system in a cellular assay, we incubated the probes with RAW 264.7 mouse macrophages for 2 h, and recorded images using confocal microscopy before and after washing the cells with buffer (Figure 3a). In the case of **6QC**, a strong specific signal was observed inside the cells after probe processing, with no significant difference between the images before and after the wash. The pHPMA probes **L-QF** and **L-FQ** exhibited behavior similar to that of **6QC**, with a limited signal present in solution and a specific signal inside the cells. Generally, both polymer probes showed lower fluorescence intensity than **6QC** after 2 h of incubation, and there was no noticeable difference based on the quencher-fluorophore arrangement. Furthermore, we included a nonquenched control probe (**nQ**) in which the fluorophore was directly tethered to the pHPMA via a short non-cleavable linker. For the **nQ** probe, a strong fluorescent signal was observed in solution after incubation, as highlighted by the difference between the images before and after the wash. This was further confirmed by the analysis of Cy5 fluorescence intensity in the medium (Figure 3b). Nevertheless, probe **nQ** was internalized by RAW 264.7 macrophages and could be detected inside the cells after washing. Probe **L-Q2:1F** generated a similar intensity of fluorescence inside the cells after the wash as the **nQ** probe, with overall reduced background fluorescence in the media (Figure 3a,b).

### Evaluation of Probe Performance *In Vivo* in a Mouse Model

**Figure 4:**
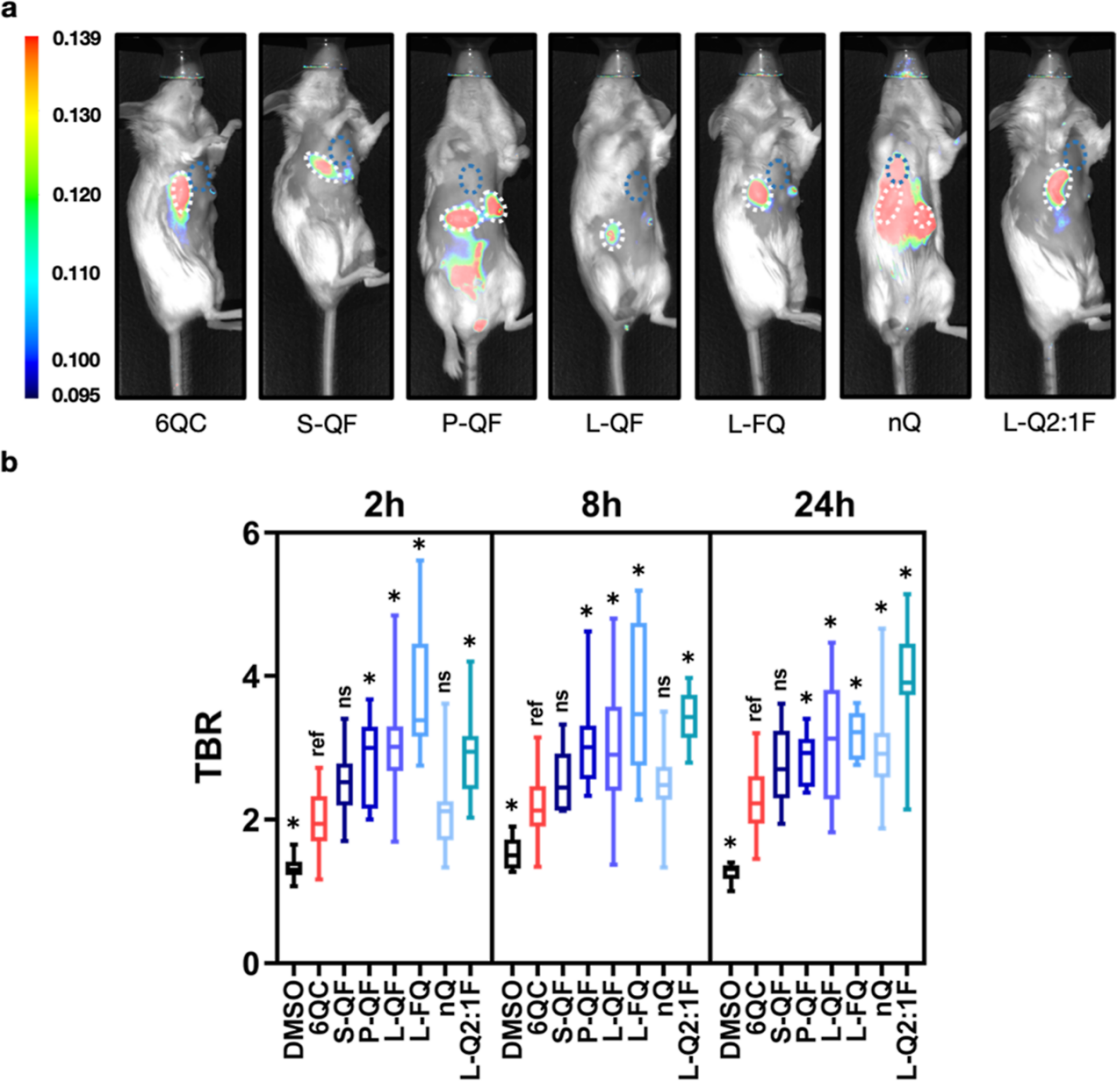
*In vivo* probe evaluation: Quantification of tumor-to-background ratio (TBR) for individual probes in live mice. (a) Representative fluorescence images showing a mouse with two breast tumors after injection of indicated probe [6.25 nmol] 24 h prior to imaging. Tumors are highlighted using a white dotted line, and background area is highlighted using blue dotted lines. Color bar shows fluorescence intensity in arbitrary units. (b) Box plots showing TBR quantification of selected probes in live mice after 2, 8, and 24 h. *N*: 3 to 10 mice, 6 to 20 tumors per condition. Images were acquired using the Pearl Trilogy small animal imaging system at the indicated time points. Statistics were calculated via the Brown–Forsythe and Welch ANOVA test with Dunnett’s multiple comparison test, asterisk represents statistical significance (*p* < 0.05), ns—not significant. Boxes extend from the 25th to the 75th percentile, with whiskers extending to minimal and maximal values. Line in the middle of the box is plotted at the median value for each sample set. All values can be found in Table S4.

**Figure 5:**
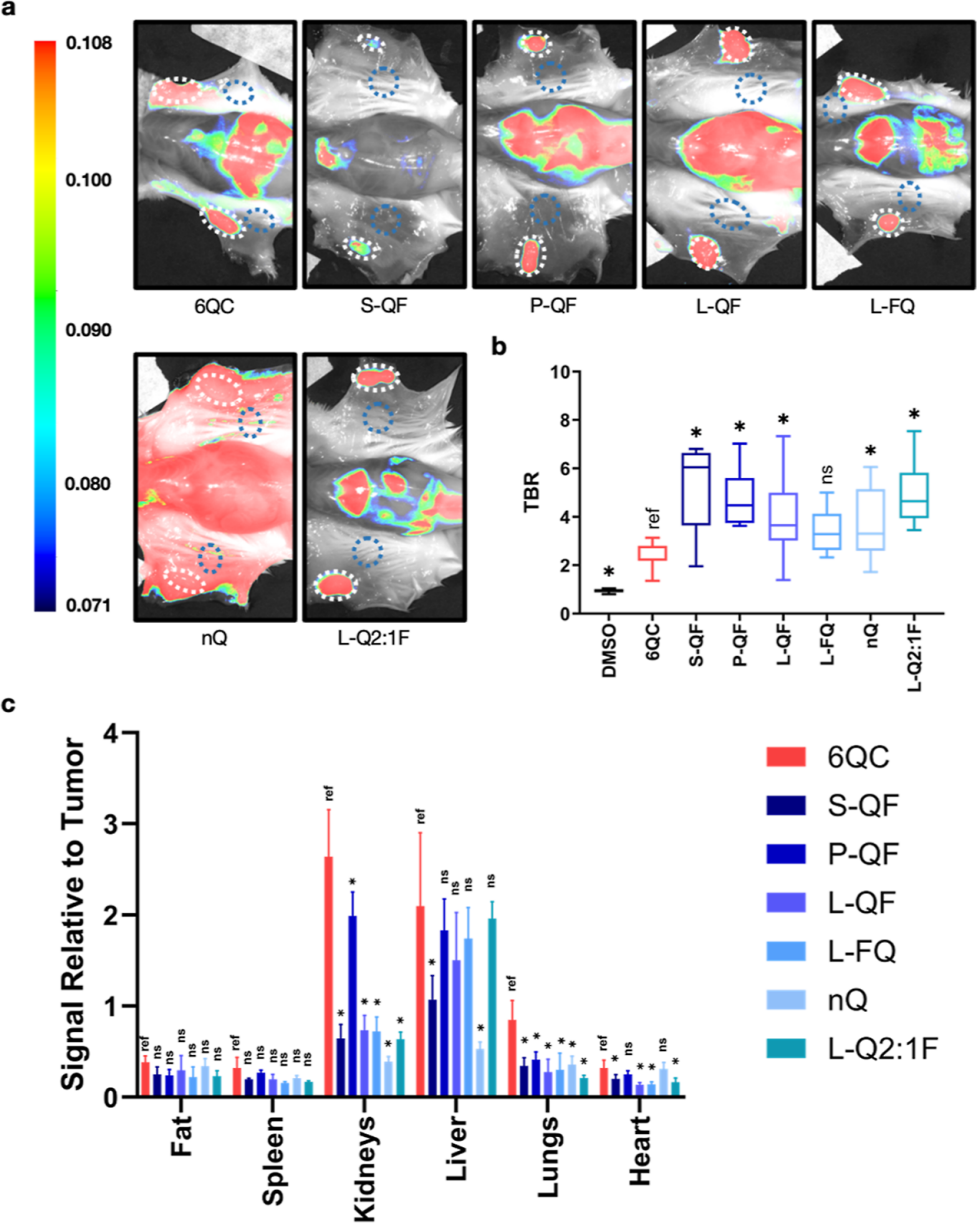
*In vivo* and *ex vivo* probe evaluations: Quantification of TBR for individual probes in splayed mice. (a) Representative fluorescence images showing a splayed mouse with two tumors after injection of indicated probe [6.25 nmol] 24 h prior to imaging. Tumors are highlighted using white dotted lines; the background area is highlighted using blue dotted lines. Color bar shows fluorescence intensity in arbitrary units. (b) Box plot showing TBR quantification of selected probes in splayed mice. (c) Bar graph showing fluorescence distribution in selected organs and tissues. *N*: 3 to 10 mice, 6 to 20 tumors per condition. Images were acquired using the Pearl Trilogy small animal imaging system. Statistics were calculated via the Brown–Forsythe and Welch ANOVA test with Dunnett’s multiple comparison test, asterisk represents statistical significance (*p* < 0.05). Boxes extend from the 25th to the 75th percentile with whiskers extending to minimal and maximal values. Line in the middle of the box is plotted at the median value for each dataset. All values can be found in Table S4.

We assessed the performance of the probes *in vivo* using an orthotopic 4T1 triple negative breast cancer mouse model. For these experiments, mice were subcutaneously injected with 4T1 cancer cells. After one week of tumor growth, the probes were intravenously administered retro-orbitally. Images were acquired at multiple time points to examine the evolution of fluorescent signal intensity and the TBR over time (Figure 4a). Tumors were easily identifiable under white light (white dotted line), and the medial chest area was selected as background (blue dotted line; concrete examples of the selection of regions of interest in live mice can be found in Figure S1). This area adjacent to the tumors was shaved in the same manner as the tumor site, and it was considered a superior option to the abdominal region, where signal from various organs could significantly influence the results. All quenched probes accumulated at the tumor site, with an efficiency similar to that of the reference probe, **6QC**. The **P-QF** probe showed an increased background fluorescent signal in the abdominal area, and the nonquenched probe **nQ** exhibited a high fluorescent signal in the surrounding tissue. Next, we determined the TBRs of all probes at three different time points (2, 8, and 24 h; Figure 4b). We selected 24 h as the final time point because, in established surgical workflows, imaging is typically conducted within this time frame after probe administration. The first three macromolecular probes (**S-QF**, **P-QF**, and **L-QF**) all possess a QF architecture, differing only in the linker structure. Generally, the TBRs of all QF architecture probes were stable over time. The **P-QF** and **L-QF** probes exhibited significantly higher TBRs than **6QC** at all studied time points, while TBRs of **S-QF** were not significantly different from **6QC** at any of the analyzed time points. At the 24 h time point, **P-QF** and **L-QF** reached TBRs of 2.9 ± 0.4 and 3.1 ± 0.8, respectively, compared to 2.3 ± 0.5 for **6QC**. The differences between **P-QF** and **L-QF** were generally small and statistically insignificant. However, the polyPro probe **P-QF** showed increased accumulation in kidneys compared to the PEG-based probes (Figure 5c); therefore, we prioritized the PEG-12 linker (L), which performed well in both *in vitro* and *in vivo* evaluations, to assess the architecture influence on probe performance.

We introduced the flipped architecture **L-FQ**, which releases the quencher instead of the fluorophore, and **L-Q2:1F** as a representative of the Q*x*:1F architecture. Similar to **L-QF**, the **L-FQ** probe exhibited significantly higher TBR than **6QC** at all analyzed time points (Figure 4b). The change in the quencher-fluorophore arrangement did not influence the TBR significantly. Interestingly, the **nQ** probe (TBR = 3.0 ± 0.8, 24 h), driven solely by the EPR effect, also provided high contrast, but required 24 h to achieve peak TBR values and establish a significant difference from **6QC**. The **L-Q2:1F** probe also showed increasing TBR values over time and reached the highest TBR (3.9 ± 0.9, 24 h) of all the probes tested.

To accurately assess the overall tumor tissue specificity of the probes, we also imaged sacrificed mice after the final time point with their skin splayed open to reveal the undersurface of the tumors (Figure 5a). In this case, we selected neighboring skin as the reference background (concrete examples of the selection of regions of interest in splayed mice can be found in Figure S2). Similar to what was observed in live mice, all quenched probes labeled tumors effectively, mostly varying in signal intensity and background fluorescence in the nontumor tissues. The QF architecture probes exhibited TBRs ranging from 3.9 ± 1.5 (**L-QF**) to 5.1 ± 1.9 (**S-QF**), compared to 2.3 ± 0.4 of **6QC** (Figure 5b). Interestingly, **S-QF** showed the highest contrast of the QF probes in this configuration. Overall, TBR values were in good correlation with the data from the live mice. The **L-Q2:1F** (TBR = 5.1 ± 1.3) and **S-QF** (TBR = 5.1 ± 1.9) probes achieved the highest TBR values in this configuration, while **L-FQ** (TBR = 3.4 ± 1.0) and **nQ** (TBR = 3.7 ± 1.4) showed slightly lower contrast compared to other polymeric probes, with **L-FQ** being the only probe, which did not achieve a significant difference from the **6QC** TBR values. Furthermore, we analyzed the biodistribution of the probes by imaging harvested organs (Figure 5c). For the fat pads and the spleen, the accumulation of **6QC** was minimal, and no further reduction was observed for the macromolecular probes. In the kidneys, accumulation was significantly reduced for all macromolecular probes, with signal intensity relative to the tumor falling below 1, compared to 2.6 ± 0.5 for **6QC**. An exception was probe **P-QF** for which the accumulation was reduced only to 2.0 ± 0.3. Interestingly, for most quenched probes, liver accumulation was not significantly different from that of **6QC** (2.1 ± 0.8), while **S-QF** (1.1 ± 0.3) and **nQ** (0.5 ± 0.1) showed a significant reduction in the liver signal. Lung accumulation was significantly reduced for all macromolecular probes compared to **6QC**, while most of them also exhibited significantly reduced accumulation in the heart.

## Discussion

Optimizing the SBR and increasing tissue specificity remain important goals in probe development. This can be achieved via the choice of an appropriate activation mechanism, optimization of activation efficiency, and finally regulation of the overall pharmacodynamics of a probe. We aimed to optimize a small molecule substrate probe by transferring it to a macromolecular core scaffold, which would allow control over probe size, positioning of fluorophore and quencher, and density of ligands on the backbone. In this study, we investigated several types of flexible or rigid linkers of varying lengths to identify an optimal configuration for cathepsin cleavage. We then investigated positioning as well as the ratios of the quenchers and the fluorophores. This allowed us to confirm that the HPMA copolymer backbone is a valuable tool for enhancing the properties of low-molecular weight quenched fluorescent probes.

Specifically, we found that linker length is important for efficient probe cleavage, while it may also affect probe biodistribution. Our data suggest that relatively long linkers are required to allow efficient cleavage by the appropriate protease, probably due to the large size of the polymer backbone (Figure 2b). The rigidness of the polyPro linker did not provide an advantage over flexible PEG-12 (Figure 2b). Interestingly, the **P-QF** probe showed significantly higher kidney accumulation than any other macromolecular probe (Figure 5c). Furthermore, since only probes **S-QF** and **nQ** exhibited significantly reduced liver accumulation, one could hypothesize that this reduction is due to the lower lipophilicity of the constructs or the absence of the quencher moiety in the case of the **nQ** probe. Additionally, the liver is well-known to contain various proteases that could process our substrate and generate signal. Therefore, it is not possible to fully understand the trends observed in this study. Nevertheless, we postulate that these findings could provide a path for further optimization of related probes. The length of the linker also has an impact on the distance between the fluorophore and the quencher in Q*x*:1F architectures, where the quencher is located on the polymer backbone (Figure 2c). Therefore, it is important to carefully select the linker length to achieve a balance between cleavage and quenching efficiency and favorable biodistribution.

The data from the cell culture models matched the results observed in the purified enzyme assay (Figure 3). Probes **L-QF** and **L-FQ** showed a high SBR due to efficient quenching. Interestingly, there was no significant difference in signal retention between probe **L-QF**, which releases the fluorophore, and probe **L-FQ**, which releases the quencher. This suggests that both the dye linked to the polymer and the free dye are retained inside the lysosomes, resulting in a durable fluorescent signal over time for both probe architectures. Probes **L-Q2:1F** and **nQ** also produced signals inside the cells but exhibited higher fluorescence in the medium. This high background due to lack of quenching is likely translated into lower TBR values for these probes *in vivo*, especially at early time points, before the free probes are cleared from the bloodstream.

Our *in vivo* experiments showed that, regardless of linker and probe structure, the use of a polymer backbone improved probe biodistribution and contrast compared to the small molecule probe **6QC**. We also found that the trends of TBR values *in vivo* correlated with the trends in enzyme cleavage rates *in vitro*, even though the TBR differences were subtle. An unexpected observation was that the **S-QF** probe, which did not show superior contrast compared to other probes in live mice, exhibited the highest TBR in the splay setup (Figure 5b). This could potentially be explained by increased overall nonspecific tissue accumulation of longer, more lipophilic linkers. Another interesting trend observed was that the iBody probes generally reached higher TBR values in the splay setup, while the values for **6QC** did not show a significant difference between the live mice and the splayed mice (Figures 4b and 5b). The most striking observation arises from the probe **L-Q2:1F** that showed poor quenching efficiency *in vitro* (Figure 2c) but, surprisingly, achieved the highest TBR *in vivo* in live mice in our dataset (Figure 4b). The discrepancy between the *in vitro* and *in vivo* results can be attributed to clearance due to blood circulation *in vivo* that reduces background coming from unquenched or poorly quenched probes over time, as evidenced by the increasing contrast of **L-Q2:1F** over time. Furthermore, due to the EPR effect, part of the signal that would be considered background *in vitro* becomes tumor-specific signal and, therefore, increases contrast.^14,15^ To verify that the performance of our probes extends beyond the EPR effect, we synthesized the **nQ** probe, which lacks the cathepsin dependence for activation of the fluorescent signal. This probe reached TBR values superior to **6QC** and comparable to other polymeric probes, but only at the 24 h time point due to the high background after injection (Figure 4b). The superior performance of quenched polymeric probes compared to **nQ** at earlier time points indicates that the contrast cannot be fully attributed to the EPR effect and, therefore, must be cathepsin-dependent. Furthermore, the significantly higher contrast provided by **L-Q2:1F** at all time points compared to **nQ** further confirms that only part of the contrast is due to the EPR effect. The superior performance of **L-Q2:1F** is also likely the result of its high cleavage rate by cathepsins *in vitro*, which is expected to translate into a high tumor signal *in vivo*. Our results demonstrate that quenching, even if only partial, can act synergistically with the enhanced uptake effects to dramatically improve contrast. Even though probe **L-Q2:1F** exhibited higher TBR values in live mice compared to the other probes, it still needs up to 24 h to develop this contrast (Figure 4b). The slow generation of contrast appears to be specific to non- or poorly quenched probes, as it is a common trait for **nQ** and **L-Q2:1F**. In contrast, probes with high quenching efficiency, such as **L-QF**, reach their peak TBR values as early as 2 h after application. This rapid signal accumulation may be more compatible with common surgical workflows.

Overall, the iBody approach provides high modularity and fine-tuning of the probe signal intensity, contrast, and kinetics. In the future, we plan to include affinity-based targeting elements in the polymer backbone to further improve biodistribution and to direct conjugates to specific tissues or cell types. Possible targets could include fibroblast activation protein (FAP),^30^ glutamate carboxypeptidase II (GCPII),^31^ or FRα.^9^ This scaffold is also ideally suited for AND-gate-type probes reported in the literature that rely on the subsequent cleavage of two orthogonal protease sequences, which increases specificity and sensitivity dramatically.^32^ AND-gate probes eliminate false positive signals caused by nonspecific cleavage of the single substrate sequence in a nontarget tissue and could help reduce the relatively high fluorescent signal in the liver and kidneys. Finally, the flexible iBody approach is also well suited for ratiometric imaging approaches that can eliminate false positive signals caused by local accumulation of contrast agents.^33^ While the EPR effect helps to increase tumor tissue accumulation of macromolecular probes, similar effects can cause probe uptake in unwanted areas, resulting in a false positive signal and, therefore, increasing the background of the probe. The ratiometric approach applied to the probes described here should help minimize this effect, resulting in an enhanced contrast. Other modifications, such as the incorporation of clinically relevant chromophores, for example, ICG or IRDye800CW,^3^ can also be applied and should not dramatically influence the physicochemical properties and biodistribution due to the primary contribution of the polymer backbone.

## Conclusions

This work demonstrates that polymers based on pHPMA provide an optimal scaffold for use in the design of optical imaging probes. The simple and modular synthesis of these polymers enables engineering of the overall ligand density as well as the architecture of dye and quenchers on the probe. Furthermore, this modular strategy enables functionalization of the probes using multiple targeting ligands. We show here that pHMPA-based probes containing a cathepsin cleavable substrate have improved properties for imaging of tumor margins compared to simple peptide scaffolds. These improvements include increased fluorescent signal and enhanced overall contrast. We believe using the pHMPA scaffold is likely to be valuable for the design and synthesis of a wide range of contrast agents for imaging cancer or other relevant markers of human diseases.

## Experimental Section

### Synthesis of Target Linker-Modified Probe Derivatives, Monomers, Copolymer Precursors, and Copolymer Conjugates

Target linker-modified probe derivatives were prepared by a combination of solid- and liquid-phase peptide chemistry. Monomers were prepared by using standard procedures available in the literature. Copolymer precursors were prepared by RAFT copolymerization and conjugated with ligands of interest via the aminolytic reaction of the polymer precursor containing TT reactive groups. A detailed description of materials, procedures, and characterizations can be found in the Supporting Information.

### Assay for Enzymatic Cleavage of Probes by Recombinant Cathepsin L

Buffer for enzymatic assay was prepared by dissolving citrate (50 mM), Triton X-100 (0.1%), and CHAPS (0.5%) in MiliQ water, and the pH was adjusted to 5.5. DTT (0.8 mg/mL) was added freshly before use. The assay was performed in a 96-well plate. Positive samples were prepared by mixing enzyme (2.2 nM, 45 μL) in assay buffer and substrate (100 or 25 μM, 5 μL) dissolved in MiliQ water. Negative samples were prepared by mixing assay buffer (45 μL) and substrate (100 or 25 μM, 5 μL). The assay was performed at 37 °C. Fluorescence values were detected by a plate reader (exc 640 nm, em 670 nm) every 45 s over 90 min. All experiments were performed in triplicates. The initial cleavage rate was evaluated as the slope of the regression curve to the initial linear phase of the kinetic curve (first 12 points). SBR was evaluated as the ratio between the fluorescence intensity of the positive sample at 90 min and the mean fluorescence intensity of the negative sample at 90 min.

### General Cell Culture Methods

All cell lines were passaged a minimum of three times after thawing before use in confocal microscopy experiments and before injection into mice. Both 4T1 cells (ATCC CRL-2539) and RAW 246.7 macrophages (ATCC TIB-71) were grown with 100 U/mL penicillin and 100 μg/mL streptomycin and with 10% fetal animal serum (FAS) supplemented into the media. 4T1 cells were cultured in Roswell Park Memorial Institute (RPMI, Corning, 10-040-CV) 1640 medium containing 2 g/L glucose and 0.3 g/mL of l-glutamine. RAW 246.7 mouse macrophages were cultured in Dulbecco’s modified Eagle’s medium (ATCC, 30-2002) with 10% FAS.

### Confocal Microscopy

RAW 246.7 cells were distributed on four-chamber microscopy dishes (Cellvis, D35C4201.5N). The next day, cells were washed, and probes were added in phenol-red free DMEM (Gibco, 21063029), 10% FAS. After incubation at 37 °C, 5% CO_2_, 2 h, cells were either directly imaged or washed 1× and incubated for 5 min with Hoechst 33342 (Tocris, 5117/50) to a final concentration 1 ng/μL prior to imaging. Images were acquired using a Zeiss LSM700 fluorescence confocal microscope with a 405 nm laser for Hoechst 33,342 staining and 639 nm for probe staining with 63× magnification (Plan-Apochromat 63×/1.40 Oil DIC M27). All settings (laser %, gain, and pinhole) were set at the beginning of the imaging and then kept constant throughout. The experiment was performed more than three times, and representative images were selected for the final figure.

### 4T1 Breast-Tumor Model

4T1 cells were prepared according to the procedure described in Supporting Information. While under isoflurane anesthesia, mice were subcutaneously injected in the third and eighth mammary fat pads of BALB/c female mice (aged 6–8 weeks; Jackson Laboratory) with 100 μL of the diluted 4T1 cells (1 × 10^5^ cells per fat pad). Once the tumors were developed, mice were injected intravenously with 6.25 nmol of probe in 100 μL of injection solution using a 28-gauge 1 mL insulin syringe into the tail vein. Post injection, the mice were noninvasively imaged using the LI-COR Pearl Trilogy imaging system at multiple time points. Mice were positioned to align the tumor being imaged in the center of the field of view. After live mice imaging, mice were euthanized using cervical dislocation under isoflurane anesthesia. Mice were then splayed and imaged. Finally, through dissection, organs were collected (liver, kidneys, spleen, lungs, and heart), as well as the primary tumors and noninjected fat pad controls. These tissues were then imaged ex vivo. Fluorescence intensity was measured using the built-in LI-COR Image Studio software. The regions of interest (tumor and background) were selected using the white light image only, with the researcher not being blinded to the condition. The mean fluorescence values in these areas were calculated and divided to obtain the TBR values. Both regions were selected using a hand drawn region of interest, with the tumor easily identifiable by the eye. The background was defined as the medial chest between and slightly superior to the tumors in live mice and as neighboring healthy tissue in splayed mice. Additional information regarding probe formulation, imaging conditions, and evaluation of obtained images can be found in the Supporting Information. All images shown were linked to display the same brightness and contrast settings for a given condition (example: 24 h images for all probes are linked).

### Statistical Analysis

Statistical analysis was performed in Excel (Microsoft, Redmond, WA, USA) and Prism (GraphPad Software, San Diego, CA, USA). Statistical significance was calculated based on the Brown–Forsythe and Welch ANOVA tests.

## Supporting Information Available

Representative images of the selection of regions of interest in live mice, representative images of the selection of regions of interest in splayed mice, characteristics of evaluated probes and the copolymer precursors, and detailed synthetic procedures, additional experimental details, materials, and methods (PDF)

## Abbreviations

FDA: the U.S. Food and Drug Administration
NIR: near-infrared
FRα: folate receptor α
TBR: tumor-to-background ratio
EPR: enhanced permeability and retention
pHPMA: *N*-(2-hydroxypropyl)methacrylamide copolymer
RAFT: reversible addition–fragmentation chain transfer
TT: thiazolidine-2-thione
Cbz: benzyloxycarbonyl
PEG: polyethylene glycol
CatL: cathepsin L
SBR: signal-to-background ratio
FAP: fibroblast activation protein
GCPII: glutamate carboxypeptidase II
min: minutes
